# 

**Published:** 1899-12-23

**Authors:** 


					TBE HOSPITAL. "The standard of highest p?rity " ? Lancet. December 23rd, 1899.
CADc?.?RY's t H ? CADc!o?RY's
? ..       MO HP TTnfl HP AT VAT TUQ TTOC-n
ABSOLUTELY PURE. NO DRUOS OR ALKALIES USED.
NO. 691J Vol. XXVII. [aNewsplper!] SATURDAY,
Dec. 23rd, 1899.
[Subscription Terms,~] pRICE TWOPENCE,

				

